# ER-Golgi dysfunction and vesicular transport alterations in rheumatoid arthritis immune cells

**DOI:** 10.3389/fimmu.2026.1811568

**Published:** 2026-05-15

**Authors:** Yangna Shi, Wei Zhang, Chenxi Li, Fanna Liu, Lianghong Yin, Dongzhou Liu, Xiaoping Hong, Zhipeng Zeng, Haitao Li, Yong Dai, Donge Tang, Wenyu Gong

**Affiliations:** 1 Department of Nephrology, The First Affiliated Hospital, Jinan University, Guangzhou, China; 2 Department of Clinical Laboratory, Peking University Shenzhen Hospital, Shenzhen, China; 3 Department of Rheumatology and Immunology, Shenzhen People’s Hospital (The First Affiliated Hospital, Southern University of Science and Technology; The Second Clinical Medical College, Jinan University), Shenzhen, China; 4 Guangdong Provincial Autoimmune Disease Precision Medicine Engineering Research Center, Shenzhen Autoimmune Disease Engineering Research Center, Shenzhen People’s Hospital (The First Affiliated Hospital, Southern University of Science and Technology; The Second Clinical Medical College, Jinan University), Shenzhen, China; 5 Clinical Medical Research Center, Shenzhen People’s Hospital (The First Affiliated Hospital, Southern University of Science and Technology; The Second Clinical Medical College, Jinan University), Shenzhen, China; 6 Future Medical Center, Shenzhen University of Advanced Technology, Shenzhen, China

**Keywords:** Endoplasmic reticulum, Golgi apparatus, protein phosphorylation, rheumatoid arthritis, vesicular transport

## Abstract

**Background:**

Rheumatoid arthritis (RA) is a chronic autoimmune disease characterized by immune cell dysfunction. The endomembrane system, consisting of the endoplasmic reticulum (ER) and Golgi apparatus (GA), plays a central role in protein synthesis and trafficking. However, the regulatory architecture of the ER-Golgi axis in RA immune cells remains incompletely understood.

**Methods:**

We performed an integrative multi-omics analysis of peripheral blood mononuclear cells (PBMCs) from 96 RA patients and 90 healthy controls (HCs). Proteomic data were obtained from a previously published study from our research group, and transcriptomic data were retrieved from the GEO database (GSE17755). Protein-protein interaction (PPI) networks were constructed using STRING and Cytoscape. Kinase activity and related signaling molecules were identified based on the functional annotations of differentially expressed proteins and phosphoproteins. Upstream transcription factor (TF) regulatory networks were built through integration with hTFtarget. Drug candidates were screened using the DSigDB database.

**Results:**

RA immune cells exhibited coordinated dysregulation of the ER-Golgi axis. Proteomics revealed downregulation of vesicular transport components (RAB1A, SEC16A) and upregulation of ER stress-related proteins (DNAJC3, SERPINH1). Phosphoproteomics identified 122 differentially phosphorylated sites, including novel hypophosphorylation of SEC16A (S1305/S1356) and decreased phosphorylation of PRKCD at T507, T295, and S664, suggesting altered PRKCD-related signaling in RA immune cells. PPI network analysis highlighted RPS3 as a dual hub linking translation and inflammatory signaling. Upstream regulatory analysis identified PML, STAT1, CBFB, and RAD21 as potential TFs, while AKT1-CK2-PRKD and TBK1-IKBKB constituted major kinase hubs. These findings indicate coordinated alterations in vesicular transport, ER stress-related processes, and inflammatory signaling in RA immune cells.

**Conclusions:**

This integrative multi-omics analysis characterizes coordinated alterations of the ER-Golgi axis in RA immune cells and highlights candidate regulatory nodes, including SEC16A phosphorylation sites, RPS3, and major kinase hubs. As a hypothesis-generating study, these findings provide a systems-level framework for understanding how endomembrane dysregulation may be associated with sustained immune activation in RA.

## Introduction

Rheumatoid arthritis (RA) is a common chronic autoimmune disease with a global prevalence of approximately 0.5%-1%, predominantly affecting middle-aged women (1). The primary pathological features of RA are synovial inflammation and joint destruction, which can lead to significant functional impairment and disability. Although the etiology and pathogenesis of RA are not yet fully elucidated, accumulating evidence indicates that immune cell dysfunction plays a critical role in the development and progression of RA (2, 3).

The endomembrane system, comprising the endoplasmic reticulum (ER) and Golgi apparatus (GA), constitutes an essential intracellular organelle network responsible for protein synthesis, folding, modification, sorting, and trafficking. The ER serves as the site for protein and lipid synthesis and is a major intracellular Ca²^+^ store (4). The GA is primarily involved in protein processing, modification, sorting, and vesicle formation and transport (5). Dysfunction of the endomembrane system is closely associated with the pathogenesis of various diseases.

In autoimmune diseases, endomembrane dysfunction may contribute to aberrant immune cell activation and excessive secretion of inflammatory factors. ER stress can activate the unfolded protein response (UPR) to maintain ER proteostasis; however, persistent ER stress may lead to apoptosis and activation of inflammatory responses (6). For instance, endoplasmic reticulum stress (ER stress) has been demonstrated to play an important role in several autoimmune diseases, including systemic lupus erythematosus (SLE) and RA (7). In RA, previous studies have shown that ER stress in synovial fibroblasts and macrophages is involved in the disease process (8). However, the systems-level architecture of the ER-Golgi axis in RA immune cells remains incompletely understood.

To address this gap, we performed an integrative multi-omics analysis of peripheral blood mononuclear cells (PBMCs) from RA patients, combining in-house proteomic and phosphoproteomic data with public transcriptomic datasets. By focusing on endomembrane system-related molecules, we characterized coordinated alterations across molecular layers and further explored upstream transcriptional and kinase regulatory networks, as well as associated drug signatures. This approach provides a systems-level view of ER-Golgi dysregulation in RA immune cells.

## Methods

### Data acquisition and deposition

The proteomic and phosphoproteomic data used in this study were generated in our laboratory using high-resolution mass spectrometry and were used for subsequent integrative analyses (9). This study enrolled 96 RA patients and 90 healthy controls (HCs). All RA patients met the 2010 American College of Rheumatology (ACR) classification criteria for RA. All participants provided written informed consent, and the study was approved by the hospital ethics committee (Approval No. LL-KY2019514). Transcriptomic data were obtained from the Gene Expression Omnibus (GEO) under accession GSE17755.

### Definition of endomembrane-related genes

Endomembrane-related genes were defined based on annotations from the Human Protein Atlas (https://www.proteinatlas.org), including genes encoding proteins localized to endomembrane-associated compartments, as well as those predicted to enter the secretory pathway or reside in membrane systems based on the presence of signal peptides and/or transmembrane domains.

### Differential proteomic and phosphoproteomic analysis

Processed protein abundance and phosphorylation site data were used to identify differentially expressed proteins and phosphorylation sites associated with the endomembrane system. Analysis between RA patients and HCs was conducted using the limma package. Differential analysis was performed for both proteomic and phosphoproteomic data using consistent criteria. Differentially expressed proteins and differentially phosphorylated sites were defined based on both fold change (FC) and statistical significance. Specifically, features with a fold change ≥ 1.5 or ≤ 0.67 and p < 0.05 were considered significantly altered.

### Protein-protein interaction, module, and hub analysis

To investigate the interaction patterns of endomembrane-related proteins, protein-protein interaction (PPI) networks were constructed based on differentially expressed proteins or proteins carrying differentially phosphorylated sites. Interaction data were retrieved from the STRING database (version 11.5, https://string-db.org), and a confidence score threshold was applied to exclude low-confidence interactions. The resulting networks were visualized in Cytoscape software (version 3.9.1) (10).

To identify densely connected functional clusters, module analysis was performed using the MCODE plugin in Cytoscape. These modules were examined to explore their involvement in biological processes relevant to the endomembrane system, including vesicular transport, protein folding, and phosphorylation-mediated signaling (11). Hub proteins were identified using the cytoHubba plugin in Cytoscape. The Maximal Clique Centrality (MCC) algorithm was used to rank proteins according to their topological importance in the network.

### Kinase-related analysis

Kinase-associated proteins were identified from the phosphoproteomic dataset and PPI network analysis (12, 13). Proteins annotated as kinases or involved in kinase-related signaling were further examined and included for subsequent analysis.

### Transcription factor regulatory network analysis

To identify upstream transcriptional regulators of endomembrane-related alterations in RA, transcriptomic, proteomic, and phosphoproteomic data were integrated. Differentially expressed genes from the transcriptomic dataset were intersected with endomembrane-related differentially expressed proteins to identify core genes showing consistent changes at both mRNA and protein levels. Experimentally validated transcription factor-target interactions for these core genes were obtained from the hTFtarget database. Candidate transcription factors were further evaluated based on their expression and/or phosphorylation changes in the proteomic and phosphoproteomic datasets. Transcription factors exhibiting altered abundance or phosphorylation levels were considered potential upstream regulators.

### Drug prediction

The DSigDB library was accessed via the Enrichr platform (https://maayanlab.cloud/Enrichr) to identify compounds associated with RA-related molecular signatures (14). Differentially expressed proteins were mapped to gene symbols, and the resulting gene set was used for enrichment analysis. Results were ranked by adjusted p-values and enrichment scores. Candidate compounds were prioritized by integrating statistical significance and biological relevance, and redundant entries were removed to simplify the results (15, 16).

### Statistical analysis

Continuous variables between two groups were compared using Student’s t-test. Statistical significance was determined using a p-value threshold of less than 0.05, with results presented as mean values plus or minus the standard deviation.

## Results

### Clinical characteristics of the study population

A total of 96 patients with RA and 90 HCs were included in this study (**Table 1**). The mean age of the RA group was 50.6 ± 15.0 years, compared with 39.0 ± 10.5 years in the HC group. The proportion of females was comparable between the two groups (85.4% in RA vs. 84.4% in HC).

**Table 1:** Clinical characteristics of patients with RA and HC.

Variable	RA (n = 96)	HC (n = 90)
Age (years)	50.6 ± 15.0	39.0 ± 10.5
Female, n (%)	82 (85.4)	76 (84.4)
Disease duration (years)	10.0 ± 8.7	NA
DAS28 score	4.2 ± 1.6	NA
SJC (range)	4 [0–28]	NA
TJC (range)	6 [0–28]	NA
ESR (mm/h)	35.6 ± 27.2	NA
CRP (mg/L)	21.2 ± 31.9	NA
RF positive, n (%)	47 (49.0)	NA
CCP positive, n (%)	48 (50.0)	NA

Values are presented as mean ± standard deviation (SD) or median [range], as appropriate.

RA, rheumatoid arthritis; HC, healthy controls; SJC, swollen joint count; TJC, tender joint count; ESR, erythrocyte sedimentation rate; CRP, C-reactive protein; RF, rheumatoid factor; anti-CCP, anti-cyclic citrullinated peptide antibody; DAS28, Disease Activity Score in 28 joints.

RA patients had a mean disease duration of 10.0 ± 8.7 years and a DAS28 score of 4.2 ± 1.6, indicating moderate to high disease activity. The median swollen joint count (SJC) was 4 (range, 0-28), and the median tender joint count (TJC) was 6 (range, 0-28). Markers of systemic inflammation were elevated in the RA group, with an erythrocyte sedimentation rate (ESR) of 35.6 ± 27.2 mm/h and C-reactive protein (CRP) levels of 21.2 ± 31.9 mg/L. In addition, 49.0% and 50.0% of RA patients were positive for rheumatoid factor (RF) and anti-cyclic citrullinated peptide (anti-CCP) antibodies, respectively. Treatment exposure was not explicitly accounted for in the current analysis, as this cohort was derived from a real-world clinical population in which treatment regimens were heterogeneous and not systematically recorded.

### Proteomic remodeling of the endomembrane system in RA immune cells

**Figure 1:**
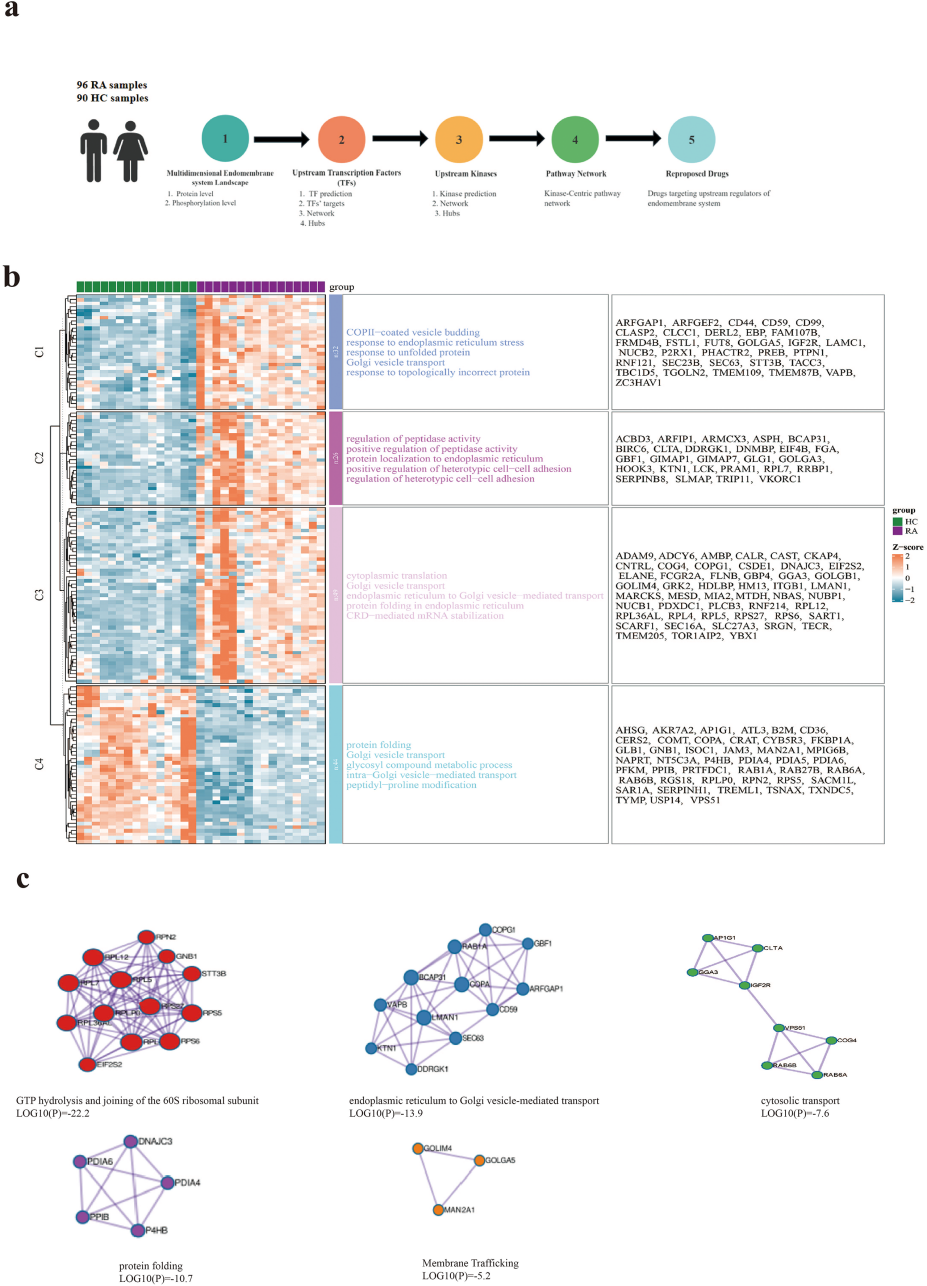
Integrated proteomic profiling reveals ER-Golgi homeostasis disruption in RA immune cells. **(a)** Study design and workflow for proteomic analysis of PBMCs from 96 RA patients and 90 HCs. **(b)** Heatmap showing ER- and Golgi-associated functional modules in RA immune cells. Color-coded Z-scores indicate relative expression levels (red: upregulated; blue: downregulated). Enrichment annotations highlight dysregulated pathways, including protein folding, vesicular transport, and translation-endomembrane coupling in RA compared with HCs. **(c)** PPI network modules of potential endomembrane-related pathways with corresponding pathway enrichment values (log10(P)). ER-to-Golgi transport module: Downregulation of potential regulators (RAB1A, SEC16A, LMAN1, COPG1; log10(P) = −13.9). ER stress and folding module: Upregulation of chaperones (P4HB, PDIA4/6) and UPR regulator DNAJC3 (log10(P) = −10.7). Translation-endomembrane coupling module: Altered interactions between ribosomal proteins (e.g., RPL7, RPS6) and ER-localized RRBP1, along with dysregulated Golgi-associated genes (membrane trafficking: log10(P) = −5.2; cytosolic transport: log10(P) = −7.6).

To systematically dissect the molecular abnormalities of the endomembrane system in RA immune cells, we performed proteomic analysis of PBMCs from 96 RA patients and 90 HCs (**Figure 1a**). Through differential protein screening and functional clustering, we generated a heatmap of ER- and Golgi-associated functional profiles (**Figure 1b**). We found that the core functional modules of the ER and Golgi in RA patients, such as protein folding and vesicular transport, exhibited pronounced expression imbalances (Z-score deviation from HC), suggesting that the disruption of endomembrane homeostasis in RA immune cells.

Integrative PPI and enrichment analyses revealed coordinated dysregulation across three interconnected layers (**Figure 1c**): (1) vesicular transport impairment, marked by downregulation of RAB1A, SEC16A, LMAN1, and COPG1; (2) ER stress activation and folding burden, characterized by upregulation of P4HB, PDIA4/6, and DNAJC3; and (3) disruption of translation-endomembrane coupling, reflected by altered ribosomal-ER interactions and Golgi-associated gene expression. Together, these alterations indicate a progressive imbalance linking transport dysfunction to inflammatory and proteostatic stress responses (17–20).

### Altered phosphoproteomic signatures of the endomembrane system in RA immune cells

**Figure 2:**
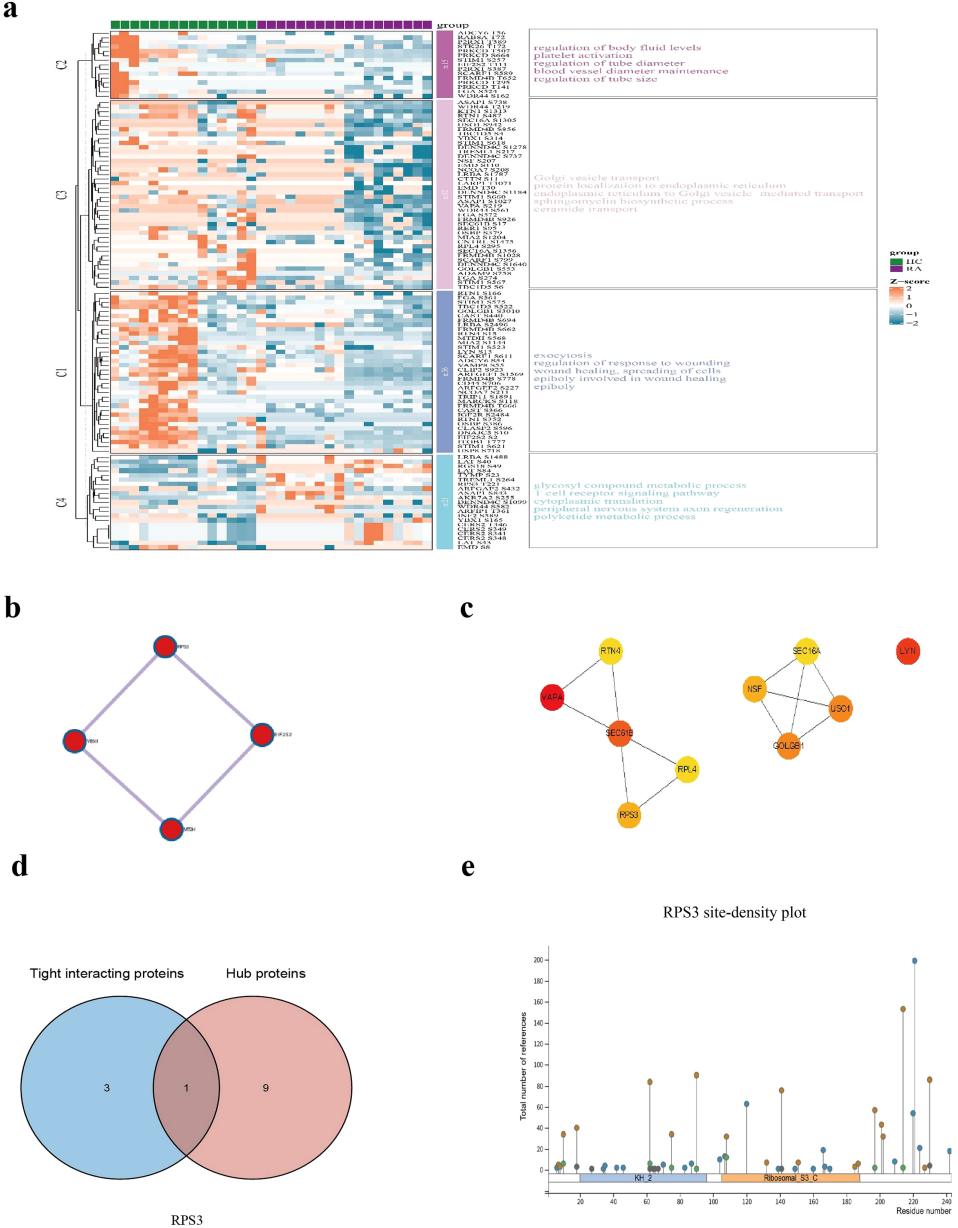
Kinase-centered phosphorylation network alterations in RA immune cells. **(a)** Heatmap of differentially phosphorylated proteins in RA immune cells with enriched biological processes annotated on the right. Z-scores represent relative phosphorylation levels (red: increased; blue: decreased), highlighting pathways associated with ER-Golgi transport, vesicle trafficking, and signal transduction. **(b)** PPI network of differentially phosphorylated proteins illustrating kinase-centered modules enriched in phosphorylation-mediated signaling pathways. **(c)** Hub protein identification using MCC topological analysis, identifying VAPA, LYN, and SEC61B as central nodes within the RA phosphorylation network. **(d)** Overlap analysis between ER stress-related proteins and hub proteins identified RPS3 as a shared regulatory node. **(e)** Phosphorylation site distribution analysis of RPS3 showing enriched modification density within specific residue regions.

Phosphorylation, a key post-translational modification, is involved in regulating protein conformation, substrate binding, and subcellular localization, thereby contributing to signal transduction and cellular homeostasis (21, 22). In RA immune cells, we used differential phosphorylation as an entry point to systematically characterize phosphoproteomic alterations associated with the endomembrane system (**Figure 2a**).

A total of 4,515 phosphorylation sites were quantified, of which 122 were identified as differentially phosphorylated. Functional annotation indicated that these sites were mainly associated with biological processes including endoplasmic reticulum (ER) stress and protein folding regulation, Golgi vesicular transport, and transmembrane signal transduction (**Figure 2a**). Notably, SEC16A exhibited decreased phosphorylation at S1305 and S1356. As a critical scaffold protein of the COPII complex, SEC16A plays an essential role in ER-to-Golgi vesicle formation and trafficking (23). The observed hypophosphorylation at these sites suggests potential alterations in vesicular transport regulation in RA. However, the functional roles of these specific phosphorylation sites in RA remain unclear and have not been previously characterized. In addition, PRKCD (PKCδ), a kinase known to participate in inflammatory signaling and apoptosis, showed significantly decreased phosphorylation at T507, T295, and S664 (24, 25). These findings indicate reduced phosphorylation at multiple regulatory sites of PRKCD, suggesting potential modulation of PKCδ-mediated signaling pathways in RA.

Other proteins with incompletely defined roles but potential significance in RA include ITGB1, RPS3, and FGA. These sites are potentially involved in the following processes: adhesion/signaling (ITGB1); translational-endomembrane coupling (RPS3); and secretion/coagulation axis (FGA). These sites may participate in the regulation of immune cell signaling, and their functional roles require further investigation (26, 27).

To further explore the functional characteristics of these differentially phosphorylated proteins, clustering analysis revealed distinct phosphorylation patterns between RA and HC samples, with proteins grouped into several clusters showing consistent trends. These clusters were primarily enriched in processes such as ER stress and protein folding, Golgi vesicular transport, and transmembrane signal transduction.

To further investigate the functional characteristics of differentially phosphorylated proteins, a PPI network was constructed (**Figure 2b**). The network revealed that these proteins were organized into several interconnected modules associated with processes such as vesicular transport, protein folding, and signal transduction.

Hub analysis based on topological features of the PPI network identified several proteins with relatively high connectivity (**Figure 2c**), suggesting their potential involvement in coordinating multiple biological processes.

By integrating module analysis and network topology, we observed that certain proteins, including RPS3, were present in multiple network modules (**Figure 2d**). In the PPI network, RPS3 interacted with several proteins, indicating its potential participation in the network structure.

The site-density plot further illustrated the distribution of phosphorylation sites along protein sequences (**Figure 2e**). For RPS3, phosphorylation sites appeared to cluster within specific residue regions, suggesting a non-random distribution pattern and potential functional relevance.

Collectively, these results highlight coordinated phosphoproteomic alterations in RA immune cells, involving key components of the ER-Golgi axis and vesicular transport machinery. Site-specific changes in SEC16A and PRKCD, together with the central network position of RPS3, point to potentially important nodes within the endomembrane-associated phosphoproteomic landscape.

### Multi-omics integration reveals an upstream TF regulatory network for the endomembrane system in RA

**Figure 3:**
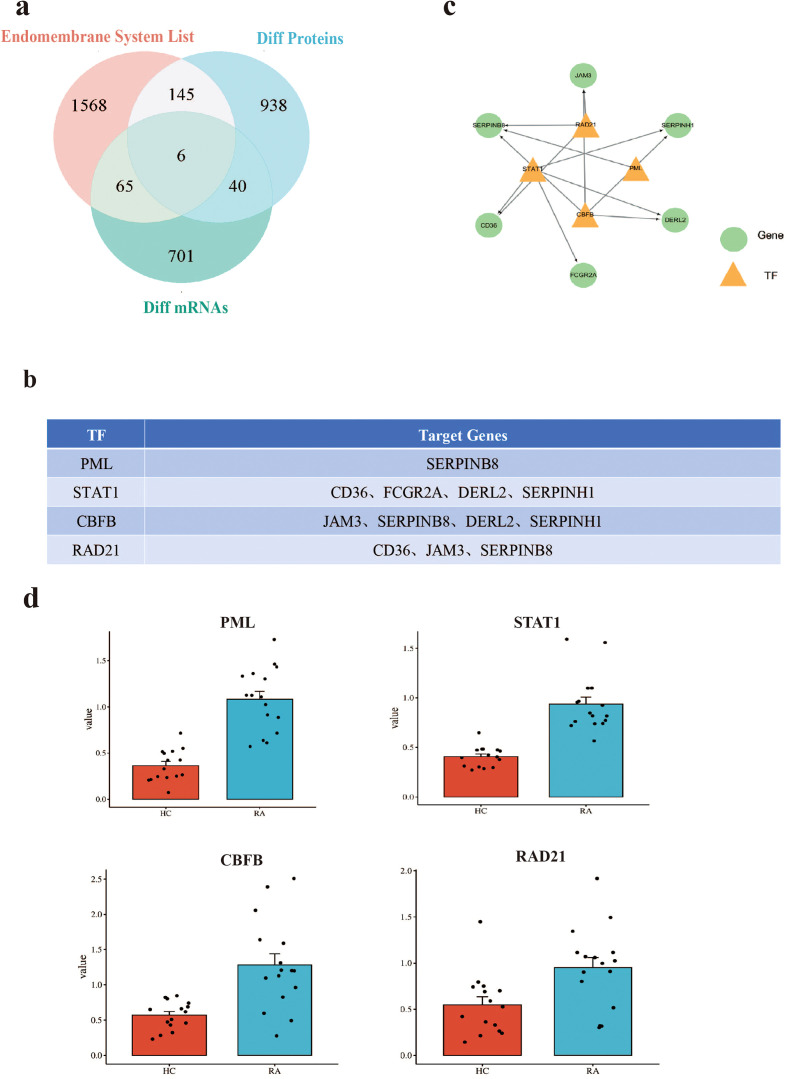
Integrated multi-omics analysis identifies upstream TFs regulation of endomembrane-related genes in RA. **(a)** Venn diagram illustrating overlap among the endomembrane system gene list (1,568 genes), differentially expressed proteins (938 proteins), and differentially expressed mRNAs (701 transcripts). Six genes (CD36, FCGR2A, JAM3, SERPINB8, DERL2, SERPINH1) exhibit consistent changes at both transcript and protein levels. **(b)** TFs and their validated target genes involved in regulating core endomembrane-associated genes. **(c)** TF-target gene regulatory network. Green circles represent TFs and orange triangles represent target genes. **(d)** Box plots showing expression levels of candidate TFs (PML, STAT1, CBFB, RAD21) in RA patients compared with healthy controls.

To elucidate the upstream regulatory mechanisms of endomembrane system genes in RA, we integrated transcriptomic, proteomic, and phosphoproteomic data to construct a TFs network. To identify endomembrane-system-related mRNAs altered in RA peripheral immune cells, we first analyzed publicly available transcriptomic datasets of RA patients and HCs from the GEO database (dataset details and processing are described in the Methods). Using thresholds of |fold change| > 1.5 and p < 0.05, we identified 71 differentially expressed genes. These genes were then intersected with our set of differentially expressed endomembrane-related proteins, yielding six overlapping candidates (**Figure 3a**). Notably, these genes (CD36, FCGR2A, JAM3, SERPINB8, DERL2, and SERPINH1) exhibited consistent alterations at both the transcript and protein levels, suggesting they may have stable functional relevance in RA.

To explore potential upstream TFs for these mRNAs in RA immune cells, we retrieved experimentally validated TFs from the hTFtarget database and further restricted the analysis to those expressed in blood cells. These candidate TFs were then intersected with the differentially expressed proteins identified in our proteomic dataset. PML, STAT1, CBFB, and RAD21 were identified among the differentially expressed proteins and were therefore retained as candidate upstream TFs (**Figures 3b–d**). These findings suggest that these TFs may be involved in the regulation of endomembrane system–related genes in RA.

### Potential signaling pathways of the endomembrane system in RA immune cells

**Figure 4:**
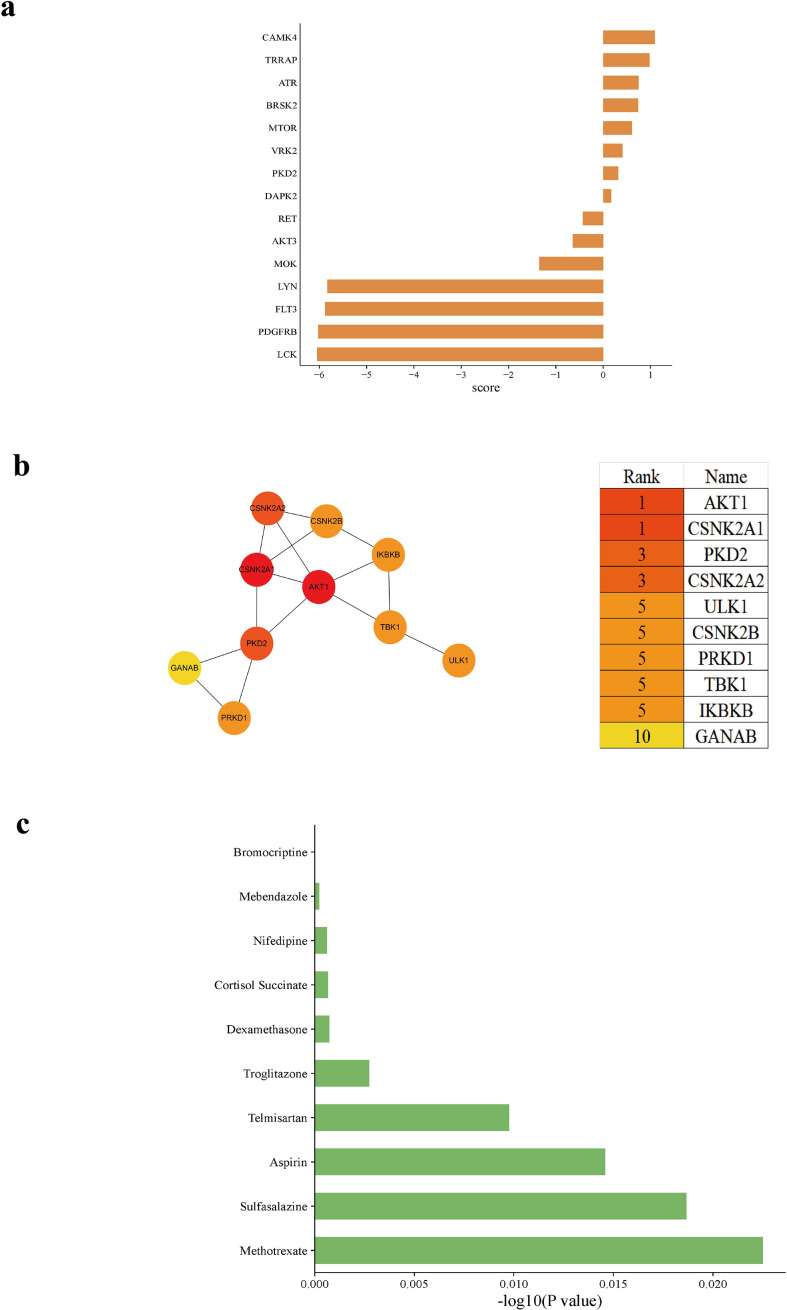
Network-based analysis of kinases and compound enrichment associated with endomembrane-related signaling in RA immune cells. **(a)** Ranking of kinases based on network-derived scores. Kinases were ordered according to their relative scores, reflecting their potential association with endomembrane-related signaling. **(b)** PPI network of selected kinases. Node color represents relative network centrality, and top-ranked kinases identified by the MCC algorithm are listed. **(c)** Compound enrichment analysis based on the input gene set using the DSigDB database. The top enriched compounds are shown according to enrichment scores (-log10 transformed).

To identify the potential upstream regulatory kinases governing the endomembrane system in RA immune cells, we estimated the activity of kinases potentially associated with the endomembrane system based on our phosphoproteomic data. Kinases including IKKβ, PKD2, CK1D, CK1E, CK2A1, CK2A2, CK2B, PKD2, TBK1, CAMK2B, AKT1, PKD3, AMPKA1, ULK2, PRKD1, and ULK1 exhibited significant activity changes in RA compared to HC group, suggesting their potential roles in disease pathogenesis (**Figure 4a**).

Among these kinases, IKKβ, AKT1, the CK2 family (CK2A1, CK2A2, CK2B), TBK1, ULK1, and AMPKA1 are widely studied in RA, where they play critical roles in regulating inflammatory signaling pathways (e.g., NF-κB, MAPK), cellular metabolism, autophagy, and abnormal synovial cell activation (28–30). In contrast, the PRKD family (PRKD1, PRKD2, PRKD3; also known as PKD1/2/3), the CK1 family (CK1D, CK1E), CAMK2B, and ULK2 have been less frequently reported in RA. They are associated with vesicular transport/membrane trafficking, calcium signaling, and specialized autophagy processes (31, 32).

Based on the PPI network and centrality ranking (**Figure 4b**), several kinases exhibited relatively high centrality scores. Among them, AKT1 and CSNK2A1 were ranked highest, followed by PKD2 and CSNK2A2. Other kinases, including ULK1, CSNK2B, PRKD1, TBK1, and IKBKB, were also identified among the top-ranked nodes. AKT1 and CK2 family members have been reported to participate in cellular processes such as metabolism and inflammatory signaling, while TBK1 and IKBKB are involved in immune and inflammatory responses. In addition, kinases such as ULK1 and TBK1 are known to regulate autophagy-related processes (33–35). Their relatively high centrality in the network suggests that they may play important roles in coordinating signaling events in RA.

### Drug prediction and target mapping

Potential pharmacologic interventions were identified using the Drug Signatures Database (DSigDB) via the Enrichr platform (https://maayanlab.cloud/Enrichr). A combined gene set was used for enrichment analysis, consisting of 71 genes representing endomembrane system–related transcriptional alterations, together with four transcription factors (PML, STAT1, CBFB, and RAD21) identified from proteomic analysis (**Figure 4c**). The inclusion of these TFs aimed to incorporate upstream regulatory components into the analysis, thereby linking transcriptional changes with their potential regulatory mechanisms. Identified compounds were further investigated by retrieving their associated gene targets from the DSigDB database. Drug–gene associations were manually curated and visualized to illustrate the relationships between candidate compounds and key genes.

## Discussion

In this study, we performed an integrative multi-omics analysis to characterize the molecular alterations of the endomembrane system (ER-Golgi axis) in RA immune cells. Proteomic analysis revealed coordinated dysregulation of vesicular transport and protein folding processes, characterized by downregulation of transport-related proteins (e.g., RAB1A and SEC16A) and upregulation of ER stress-associated proteins (e.g., DNAJC3 and SERPINH1). Phosphoproteomic profiling further identified differentially phosphorylated sites involved in ER stress, Golgi transport, and signal transduction, including decreased phosphorylation of SEC16A (S1305/S1356) and reduced phosphorylation of PRKCD at T507, T295, and S664.

Network-based analyses indicated that proteins involved in translation and membrane-associated processes, such as RPS3, were present in multiple network modules. Integration with transcriptomic data identified potential upstream regulatory factors, including PML, STAT1, CBFB, and RAD21, which were differentially expressed at the protein level. In addition, kinase-centered network analysis suggested that signaling pathways involving AKT1, CK2 family members, PRKD, TBK1, and IKBKB may participate in the regulation of these processes.

**Figure 5:**
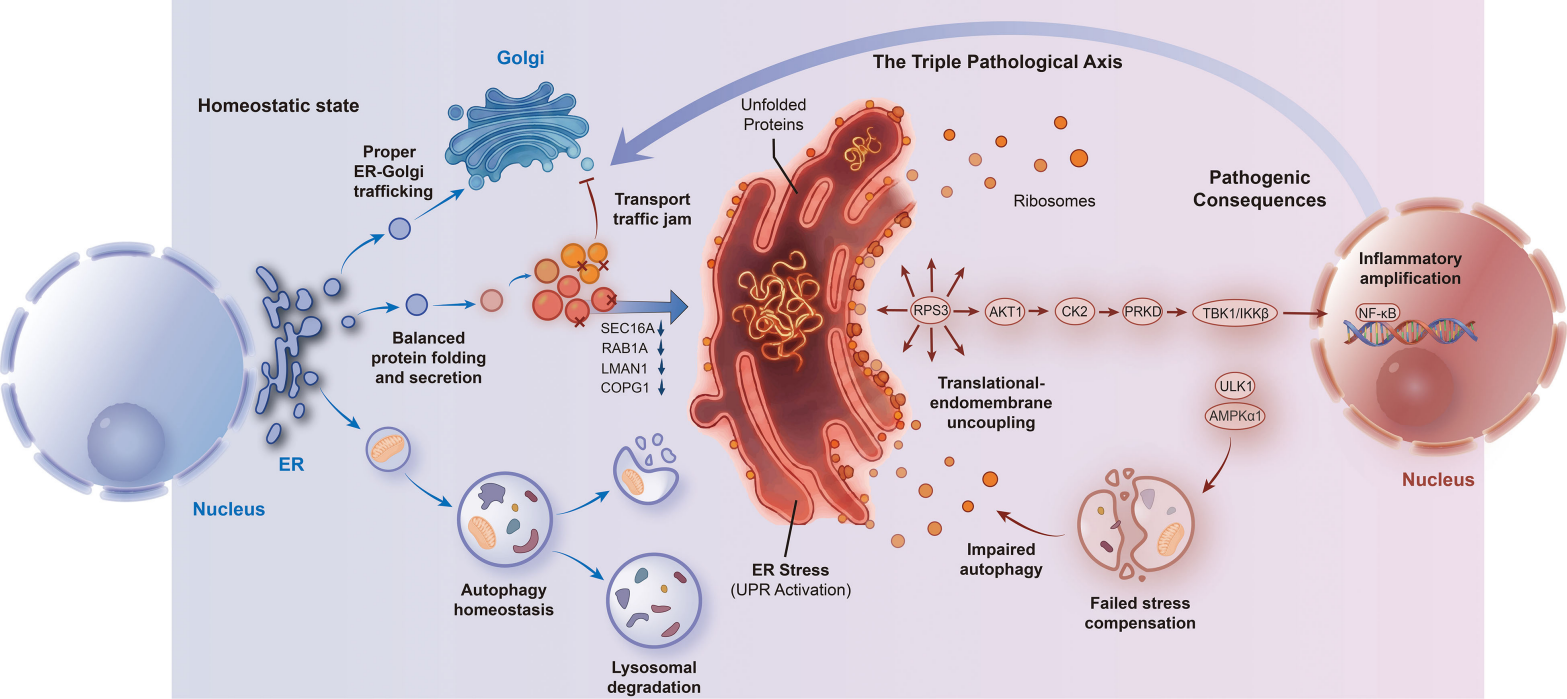
Multi-omics summary of ER–Golgi dysregulation in RA PBMCs. Proteomic and phosphoproteomic alterations in vesicular transport, ER stress, and signaling are summarized. RPS3 is shown as a shared network node, and candidate regulators are indicated. Blue arrows denote protein abundance changes, and red arrows denote phosphorylation changes. This schematic summarizes observed and inferred findings and does not imply a causal pathway.

Together, these findings suggest that coordinated alterations in vesicular transport, ER stress response, and inflammatory signaling may contribute to endomembrane system dysfunction in RA immune cells, providing a systems-level perspective for understanding RA pathogenesis (**Figure 5**).

In our results, widespread imbalances in the expression of ER- and Golgi-associated proteins were observed in RA immune cells. Aberrant expression of ER folding chaperones (e.g., P4HB, PDIA4, DNAJC3) and Golgi assembly factors (e.g., LMAN1, COPG1, TRIP11) suggests a breakdown in the coordination between protein folding and vesicular transport (17, 20, 36). The downregulation of RAB1A and SEC16A reflects impaired COPII vesicle generation, a process that may constitute an initiating event for secretory dysfunction and inflammatory amplification in RA immune cells (18, 23). This finding may expand the pathological paradigm of RA from the traditional view of “excessive cytokine secretion” to one driven by aberrant inflammatory amplification resulting from intracellular transport blockade, thereby suggesting a possible explanation for the disease’s initiating mechanisms.

Our phosphoproteomic results unveiled RA-specific regulatory hierarchies. The hypophosphorylation of SEC16A at S1305/S1356 and the reduced phosphorylation of PRKCD at T507, T295, and S664 represent site-specific phosphoproteomic alterations identified in this study. These findings suggest potential cross-talk between ER-Golgi vesicle formation and inflammatory signaling in RA immune cells. Given the known involvement of PKCCD in inflammatory and stress-related pathways, the altered phosphorylation pattern of PRKCD may reflect perturbation of PRKCD-related signaling rather than definitive activation or inhibition. The functional consequences of these site-specific changes require further experimental validation (19). Furthermore, aberrant phosphorylation of RPS3 highlights its position at the intersection of translation and endomembrane stress. Given its dual identity as both a ribosomal component and a regulator of the NF-κB complex, RPS3 may serve as a potential bridge linking imbalances in protein synthesis to immune activation (27, 37). This discovery offers a novel molecular explanation for the persistent inflammation characteristic of RA.

In this study, our integrative multi-omics analysis provides evidence suggesting that dysregulation of the endomembrane system, particularly along the ER-Golgi axis, may contribute to immune-related processes in RA. Previous studies have highlighted functional links between PTM proteins, EVs and immune activation in RA. Our findings extend this framework by suggesting that alterations in endomembrane-associated pathways may underlie these processes. In particular, the coordinated changes observed across transcriptomic, proteomic, and phosphoproteomic levels point to potential disruptions in vesicular trafficking, protein processing, and signaling regulation (38–40). Given that EV biogenesis and cargo transport are highly dependent on ER-Golgi-mediated vesicular trafficking, our results support a hypothetical model in which endomembrane dysfunction may influence the generation and release of PTM-containing antigens, thereby contributing to immune activation and loss of tolerance in RA. However, these mechanistic links are inferred from integrative analysis and require further experimental validation.

Among the enriched compounds, methotrexate and telmisartan appear particularly noteworthy. Methotrexate, which showed the strongest enrichment signal, is an established disease-modifying antirheumatic drug (DMARD) (38, 41). Its identification in our analysis supports the biological relevance of the RA-associated molecular signature captured in this study. In contrast, telmisartan may represent a potentially interesting repurposing candidate. Beyond its known role in metabolic and PPAR-related regulation, its enrichment in our dataset suggests that pathways linked to metabolic stress and inflammatory activation may intersect with the endomembrane abnormalities observed in RA immune cells. From a translational perspective, methotrexate primarily serves as an internal validation of the analytical framework, whereas telmisartan may be of greater interest for future investigation as an adjunctive or repurposed therapeutic strategy (42, 43). In particular, it may have potential value in RA patients with prominent metabolic dysregulation or persistent inflammatory activity. However, these associations are inferred from transcriptional signature overlap rather than direct drug-target validation, and therefore should be interpreted cautiously pending further functional and clinical studies.

This study should be interpreted in light of several limitations. First, the present work is primarily descriptive and hypothesis-generating in nature. Although the integrated transcriptomic, proteomic, and phosphoproteomic analyses provide convergent evidence supporting SEC16A phosphorylation, RPS3, and several kinases (including AKT1, CK2 family members, PRKD, TBK1, and IKBKB) as candidate regulators of ER-Golgi dysfunction in RA immune cells, this study does not by itself establish direct causality or definitive molecular mechanism. Second, the analyses were performed in PBMCs, which capture systemic immune alterations but do not resolve cell type-specific effects or directly address tissue-context mechanisms in the synovium. Third, orthogonal validation and functional perturbation experiments, including independent cohort validation, site-directed mutagenesis, and kinase/pathway intervention studies, are still needed. In addition, as this study was conducted in a real-world clinical cohort where treatment exposure was not systematically controlled or modeled, the potential influence of medications on the observed molecular signatures cannot be fully excluded, and future validation in treatment-naïve or well-annotated cohorts will be important.

## Conclusions

In conclusion, this study characterizes coordinated ER-Golgi dysfunction in RA immune cells and highlights potential regulatory nodes, including SEC16A, RPS3, and major kinase hubs, that link vesicular transport impairment with inflammatory signaling. These findings support a mechanistic framework in which disruption of endomembrane homeostasis contributes to sustained immune activation in RA and provide a foundation for further experimental validation.

## Data Availability

Publicly available datasets were analyzed in this study. The phosphoproteomics data generated in this study have been deposited in the ProteomeXchange Consortium via the PRIDE repository under accession number PXD025559: https://www.ebi.ac.uk/pride/archive/projects/PXD025559. Visualization of MS/MS spectra is available via MS-Viewer at: https://msviewer.ucsf.edu/prospector/cgi-bin/mssearch.cgi?report_title=MS-Viewer&search_key=wvw7pyeqyq&search_name=msviewer. The proteomics datasets were generated in our laboratory and are available upon reasonable request.
